# Comparative Evaluation of Doppler Sonography and Magnetic Resonance Angiography in Assessing Extracranial Carotid and Vertebral Arteries in Patients With Stroke

**DOI:** 10.7759/cureus.100110

**Published:** 2025-12-26

**Authors:** Harish N, Ravi Kumar Yeli, Amruth V C, Suresh Kanamadi

**Affiliations:** 1 Department of Radiology, Shridevi Institute of Medical Sciences and Research Hospital, Tumakuru, IND; 2 Department of Radiology, Bijapur Lingayat District Educational (BLDE) Deemed to be University (DU) Shri B. M. Patil Medical College, Hospital and Research Centre, Vijayapura, IND

**Keywords:** doppler sonography, extracranial carotid artery, magnetic resonance angiography (mra), stroke, vertebral artery

## Abstract

Objective: This study aimed to evaluate the performance of Doppler sonography in identifying extracranial vascular abnormalities in patients with stroke and to compare its findings with those obtained from magnetic resonance angiography (MRA).

Methodology: This observational study included 150 patients with stroke or transient ischemic attack (TIA) who presented to Shri B.M. Patil Medical College between November 2019 and September 2022. All patients underwent color Doppler ultrasonography using a Philips HD-11 XE machine (Koninklijke Philips N.V., Amsterdam, Netherlands) with a 4-11 MHz linear probe to evaluate the carotid and vertebral arteries for stenosis, plaque formation, and flow irregularities. MRA was performed on a GE 1.5 Tesla SIGNA MRI scanner (GE Healthcare, IL, USA) using 3D time-of-flight (TOF) and gadolinium-enhanced sequences. Imaging findings from both modalities were compared using paired proportion statistical analysis.

Results: The mean age of participants was 60.66 ± 10.91 years, comprising 126 males (84%) and 24 females (16%). The internal carotid artery (ICA) and vertebral artery were the most frequently affected vessels, demonstrating abnormalities in 84 (56%) and 78 (52%) cases, respectively, on both Doppler and MRA. Bulb involvement was observed in 21 (14%) cases on Doppler and 15 (10%) on MRA. Collateral formation was most frequently noted on MRA in the vertebral artery (51 (34%)) and ICA (45 (30%)), whereas Doppler detected none. Complete occlusion and thrombosis were each most commonly observed in the ICA and vertebral arteries (78 (52%) cases).

Conclusions: Doppler sonography is a reliable bedside screening tool for detecting significant occlusive disease in patients with stroke. However, MRA offers superior resolution, particularly for assessing high-grade stenosis and collateral circulation. Together, they serve as complementary diagnostic modalities, and their combined use may enhance stroke evaluation and support more effective therapeutic decision-making.

## Introduction

Stroke remains a leading cause of morbidity and mortality worldwide and represents a significant global public health concern. It is defined as a sudden-onset neurological deficit caused by a vascular event, including cerebral infarction, intracerebral hemorrhage, or subarachnoid hemorrhage. Ischemic strokes account for approximately 80-87% of all strokes and are most commonly caused by atherosclerotic disease or embolic events affecting the extracranial carotid and vertebral arteries [1]. These arteries are critical conduits of cerebral blood flow, and pathological changes such as stenosis or dissection can substantially increase stroke risk, particularly among younger patients [2].

Early identification of vascular abnormalities in the extracranial arteries is pivotal in acute stroke evaluation and secondary prevention. Among imaging modalities, color Doppler sonography has emerged as a non-invasive, widely available, and cost-effective tool that provides real-time assessment of vascular flow dynamics, arterial patency, and the degree of stenosis [3]. However, its diagnostic utility may be limited by operator dependency and anatomical challenges [4].

In contrast, magnetic resonance angiography (MRA) is a high-resolution, non-invasive technique that allows detailed visualization of both extracranial and intracranial vasculature without the use of ionizing radiation or iodinated contrast agents [5], making it particularly advantageous for patients with renal impairment or contrast allergies [6].

While both modalities are routinely used in stroke imaging, their comparative accuracy and clinical utility in evaluating extracranial carotid and vertebral artery disease remain areas of ongoing investigation. This is particularly relevant in resource-limited settings where access to advanced imaging such as MRA may be restricted.

This study aims to evaluate the diagnostic utility of Doppler sonography in assessing the extracranial carotid and vertebral arteries in patients presenting with stroke and to compare its findings with those obtained from MRA. The comparison seeks to inform clinicians about the reliability, accessibility, and limitations of each modality, thereby supporting appropriate modality selection based on clinical context and available resources.

## Materials and methods

Study design and setting

This hospital-based observational study was conducted at Shri B. M. Patil Medical College, Vijayapura, between November 2019 and September 2022. During this period, patients presenting with clinical signs and symptoms of stroke or transient ischemic attack (TIA) were enrolled. After obtaining informed consent and performing clinical evaluations, 150 patients were included. Shri. B.M Patil Medical College, Hospital and Research Centre issued approval IEC/No-131/2015. Each patient underwent a detailed medical history and neurological examination. Risk factors such as hypertension, diabetes mellitus, smoking, and ischemic heart disease were documented.

Inclusion and exclusion criteria

Patients aged 18 years and above who presented with clinically suspected acute ischemic stroke or TIA, diagnosed based on neurological assessment by a consultant neurologist, were included in the study. All enrolled patients underwent both color Doppler ultrasound and MRA within a defined time window following symptom onset to ensure comparability of imaging findings. Patients with known intracranial vascular malformations, previous carotid endarterectomy or stenting, contraindications to MRI, or those unable to cooperate during imaging were excluded.

Sample size calculation

The sample size was estimated based on an anticipated prevalence of extracranial carotid and vertebral artery stenosis in patients with ischemic stroke. Assuming a prevalence of 30%, with a 95% confidence interval and 8% absolute precision, the minimum required sample size was calculated to be 126 using the formula:

\[ n = \frac{Z^{2} \times p \times (1 - p)}{d^{2}} \] where $Z = 1.96$, $p = 0.30$, and $d = 0.08$. A final sample size of $n = 150$ patients was targeted and achieved to account for potential dropouts or exclusions.

Doppler ultrasound evaluation

A color Doppler examination of the extracranial carotid and vertebral arteries was performed using a Philips HD-11 XE ultrasound machine (Koninklijke Philips N.V., Amsterdam, Netherlands) equipped with a 4-11 MHz high-definition linear array probe. All examinations were conducted by trained radiologists with experience in vascular ultrasonography, following a standardized scanning protocol. To ensure consistency, predefined criteria were used for image acquisition and interpretation, and images were reviewed in accordance with departmental quality assurance practices.

All Doppler assessments were conducted at a fixed insonation angle of 60°. Patients were examined in the supine position with the neck extended and rotated away from the scanned side. The examiner was positioned on the patient’s right side. Transducer positions included lateral, posterolateral, and anterior orientations to obtain longitudinal and transverse views. Parameters recorded included peak systolic velocities (PSV) of the internal carotid artery (ICA)/common carotid artery (CCA) velocity ratios, plaque morphology, and spectral broadening patterns.

The examination began with a longitudinal survey of the carotid arteries, identifying the CCA at the clavicle and tracing it cephalad to the bifurcation. The internal and external carotid arteries were distinguished, and the ICA was followed as cranially as possible. All suspected plaques were evaluated for extent, echotexture, and degree of luminal narrowing. Findings were reconfirmed using anterior and transverse scanning planes to provide a three-dimensional perspective. The severity of stenosis was assessed by correlating B-mode imaging with Doppler velocity criteria.

MRA protocol

MRA was performed on a GE 1.5-Tesla SIGNA 16-channel MRI system (GE Healthcare, IL, USA) with gradient overdrive capability, providing a slew rate of 120 mT/sec and a peak amplitude of 33 mT/m. A circularly polarized phased-array neck coil (4 × 2) was used for optimal image acquisition. After obtaining axial, coronal, and sagittal localizers, two angiographic sequences were performed: 3D time-of-flight (TOF) and 3D gadolinium-enhanced MRA.

The 3D TOF technique, selected for its superior spatial resolution and reduced flow artifacts, comprised seven slabs with 32 axial sections each, 1.41 mm thick, using a TR/TE of 39/7, a flip angle of 25°, and a matrix size of 160 × 256 within a 250 × 250 × 90 mm field of view. The scan was centered on the carotid bifurcation. Subsequently, contrast-enhanced MRA was performed using a spoiled gradient-recalled echo sequence with a TR of 1.5 msec, TE of 1.0 msec, and a flip angle of 30°. Forty-two partitions of 2 mm thickness were acquired within four seconds, with a 300 × 225 mm field of view and a matrix size of 320 × 160. Fifteen milliliters of gadodiamide contrast was injected manually at 2-3 mL/sec into an antecubital vein, followed by a 15 mL saline flush. No bolus timing or breath-holding was used.

Image processing and interpretation

Images were processed using a maximum-intensity projection (MIP) algorithm. For TOF sequences, targeted MIP reconstructions displaying 13 projections at 14° intervals were generated. In gadolinium-enhanced studies, the pre-contrast mask image was subtracted from the arterial-enhanced sequence to isolate the arterial signal. In cases of venous enhancement, appropriate subtraction adjustments were applied to optimize arterial visualization. All images were reviewed independently by radiologists blinded to the Doppler findings.

Statistical analysis

All collected data were entered into Microsoft Excel (Microsoft Corporation, Redmond, Washington, United States) and analyzed using IBM SPSS Statistics for Windows, Version 25 (released 2017; IBM Corp., Armonk, New York, United States). Continuous variables were summarized as means and standard deviations, while categorical variables were presented as frequencies and percentages. Comparisons between Doppler and MRA findings were made using chi-square tests or Fisher’s exact test, as appropriate. A p-value of <0.05 was considered statistically significant.

## Results

Table 1 summarizes the demographic and clinical characteristics of the study participants. The mean age was 60.66 ± 10.91 years. Of the 150 participants, 126 (84%) were male, and 24 (16%) were female. Regarding clinical history, three (2%) participants had diabetes mellitus, 60 (40%) had hemiparesis, 12 (8%) had a history of cerebrovascular accident (CVA), 57 (38%) reported stroke, and 18 (12%) reported general weakness.

**Table 1:** Baseline characteristics of study participants CVA: cerebrovascular accident

Variables	Total number N (%)
Mean age (in years)	60.66 ± 10.91
Gender	
Male	126 (84%)
Female	24 (16%)
Clinical history	
Diabetes mellitus	3 (2%)
Hemiparesis	60 (40%)
CVA	12 (8%)
Stroke	57 (38%)
Weakness	18 (12%)

Overall, vascular abnormalities, including stenosis and occlusion, were most frequently identified in the vertebral artery and the ICA, with involvement detected in 78 (52%) and 84 (56%) cases, respectively, by both Doppler sonography and MRA. Carotid bulb involvement was observed in 21 (14%) cases on Doppler and 15 (10%) on MRA. Pathology of the CCA was uncommon, identified in three (2%) cases across both modalities, while no abnormalities were detected in the external carotid artery (ECA). Following this overall distribution, stenotic lesions were further classified by severity. The most frequent grade of stenosis was 70-79%, predominantly involving the carotid bulb (6 (4%)) and ICA (3 (2%)) on both Doppler and MRA. Mild stenosis (16-49%) was observed exclusively in the carotid bulb, affecting 6 (4%) cases on Doppler and 3 (2%) on MRA. Moderate (50-69%) and severe (80-89%) stenosis were confined to the ICA and carotid bulb, with frequencies ranging from 3 (2%) to 6 (4%) depending on the imaging modality. No graded stenosis was identified in the CCA, ECA, or vertebral arteries (Table 2).

**Table 2:** Comparison of Doppler and MR angiography findings across arterial segments CCA: common carotid artery; BULB: carotid bulb; ECA: external carotid artery; ICA: internal carotid artery

Parameters	Pathology				
	CCA	BULB	ECA	ICA	Vertebral
Total pathology					
Doppler	3 (2%)	21 (14%)	0 (0%)	84 (56%)	78 (62%)
MR Angiography	3 (2%)	15 (10%)	0 (0%)	84 (56%)	78 (62%)
16-49% stenosis					
Doppler	0 (0%)	6 (4%)	0 (0%)	0 (0%)	0 (0%)
MR Angiography	0 (0%)	3 (2%)	0 (0%)	0 (0%)	0 (0%)
50-69% stenosis					
Doppler	0 (0%)	6 (4%)	0 (0%)	3 (2%)	0 (0%)
MR Angiography	0 (0%)	0 (0%)	0 (0%)	0 (0%)	0 (0%)
70-79% stenosis					
Doppler	0 (0%)	6 (4%)	0 (0%)	3 (2%)	0 (0%)
MR Angiography	0 (0%)	6 (4%)	0 (0%)	3 (2%)	0 (0%)
80-89% stenosis					
Doppler	0 (0%)	0 (0%)	0 (0%)	0 (0%)	0 (0%)
MR Angiography	0 (0%)	6 (4%)	0 (0%)	3 (2%)	0 (0%)

Complete occlusion on Doppler and MRA was most frequently observed in the ICA and vertebral artery, respectively, in 78 (52%) cases. The CCA showed complete occlusion in only 1 (0.66%) case, while no complete occlusion was noted in the bulb or ECA on either imaging modality (Table 3).

**Table 3:** Comparison of complete occlusion detected by Doppler and MR angiography CCA: common carotid artery; BULB: carotid bulb; ECA: external carotid artery; ICA: internal carotid artery

Complete occlusion	Doppler	MR angiography
CCA	1 (0.66%)	1 (0.66%)
BULB	0 (0%)	0 (0%)
ECA	0 (0%)	0 (0%)
ICA	78 (52%)	78 (52%)
Vertebral	78 (52%)	78 (52%)

Thrombosis was most frequently observed in the ICA and vertebral artery, each in 78 (52%) cases, on both Doppler and MRA. The CCA showed thrombosis in 3 (2%) cases, while no thrombosis was detected in the bulb or ECA on either imaging modality (Table 4).

**Table 4:** Comparison of thrombosis detected by Doppler and MR angiography CCA: common carotid artery; BULB: carotid bulb; ECA: external carotid artery; ICA: internal carotid artery

Thrombosis	Doppler	MR angiography
CCA	3 (2%)	3 (2%)
BULB	0 (0%)	0 (0%)
ECA	0 (0%)	0 (0%)
ICA	78 (52%)	78 (52%)
Vertebral	78 (52%)	78 (52%)

On Doppler examination, collateral formation was not observed in any arteries. However, on MRA, collaterals were most frequently seen in the vertebral artery in 51 (34%) cases, followed by the ICA in 45 (30%) cases, and the CCA in 3 (2%) cases. No collaterals were detected in the bulb or ECA on either modality. Regarding the location of stenosis, both Doppler and MRA demonstrated stenosis most commonly at the bulb region in 21 (14%) cases, followed by the ICA in 6 (4%) cases. Using either imaging technique, no stenosis was noted in the CCA, ECA, or vertebral arteries (Table 5).

**Table 5:** Comparison of collateral circulation and location of stenosis detected by Doppler and MR angiography CCA: common carotid artery; BULB: carotid bulb; ECA: external carotid artery; ICA: internal carotid artery

Variables	Doppler N (%)	MR angiography N (%)
Collaterals		
CCA	0 (0%)	3 (2%)
BULB	0 (0%)	0 (0%)
ECA	0 (0%)	0 (0%)
ICA	0 (0%)	45 (30%)
Vertebral	0 (0%)	51 (34%)
Location of stenosis		
CCA	0 (0%)	0 (0%)
BULB	21 (14%)	21 (14%)
ECA	0 (0%)	0 (0%)
ICA	6 (4%)	6 (4%)
Vertebral	0 (0%)	0 (0%)

Among the 150 patients assessed, ICA involvement was most frequently observed, with 84 (56%) cases showing pathology on both Doppler and MRA. Of these, right-sided ICA involvement was seen in 48 (32%), left-sided in 27 (18%), and bilateral in 9 (6%) cases on both modalities. Vertebral artery involvement was also common, detected in 78 (52%) cases with identical laterality on both Doppler and MRA: right-sided in 36 (24%), left-sided in 21 (14%), and bilateral in 21 (14%). Bulb pathology was present in 21 (14%) patients on Doppler, including 3 (2%) bilateral, 12 (8%) right-sided, and 6 (4%) left-sided; on MRA, 3 (2%) were bilateral, 9 (6%) right-sided, and 3 (2%) left-sided. The CCA showed left-sided pathology in 3 (2%) cases on both modalities, with no right-sided or bilateral involvement. No pathology was observed in the ECA on either side or bilaterally by Doppler or MRA (Table 6).

**Table 6:** Laterality of total pathologies (stenosis + occlusion) detected by Doppler and MR angiography CCA: common carotid artery; BULB: carotid bulb; ECA: external carotid artery; ICA: internal carotid artery

Affected side (total pathologies stenosis + occlusion)	Total no. of patients	Doppler			MR angiography		
		Both	Right	Left	Both	Right	Left
CCA	150	0 (0%)	0 (0%)	3 (2%)	0 (0%)	0	3 (2%)
BULB	150	3 (2%)	12 (8%)	6 (4%)	3 (2%)	9 (6%)	3 (2%)
ECA	150	0 (0%)	0 (0%)	0 (0%)	0 (0%)	0 (0%)	0 (0%)
ICA	150	9 (6%)	48 (32%)	27 (18%)	9 (6%)	48 (32%)	27 (18%)
Vertebral	150	21 (14.0%)	36 (24.0%)	21 (14.0%)	21 (14.0%)	36 (24.0%)	21 (14.0%)

**Figure 1:**
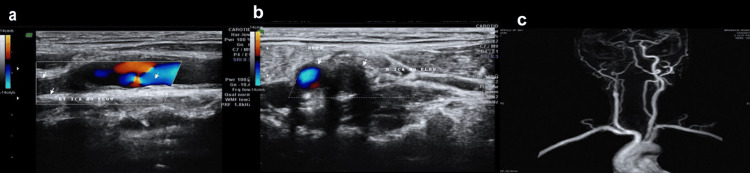
Imaging findings of right internal carotid artery occlusion a: color Doppler ultrasonography shows no flow in the right internal carotid artery (ICA) (arrows); b: longitudinal Doppler view confirming the occluded segment of the right ICA with no detectable flow (arrows); c: magnetic resonance angiography (MRA) demonstrating complete occlusion of the right ICA with preserved flow in the contralateral carotid and vertebrobasilar circulation.

Carotid Doppler ultrasonography demonstrated complete occlusion of the right ICA. On color Doppler imaging, the flow signal was absent within the right ICA lumen, while the common carotid artery showed preserved antegrade flow, suggesting that the occlusion site was distal to the bifurcation. The corresponding grayscale images revealed a hypoechoic area with no detectable intraluminal flow. MRA further confirmed the absence of flow-related enhancement in the right ICA, consistent with total occlusion. The left carotid and vertebrobasilar systems were patent with standard flow patterns (Figure 1).

**Figure 2:**
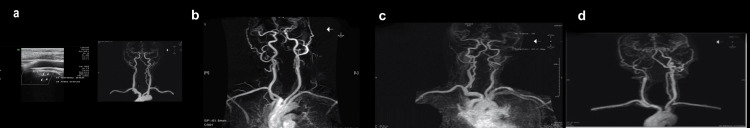
Imaging findings demonstrating right internal carotid artery occlusion a: combined Doppler ultrasound and MRA images show no flow in the right internal carotid artery (ICA); b: MRA coronal projection confirming complete occlusion of the right ICA with intact left-sided and posterior circulation (arrow); c: sagittal view of MRA illustrating non-visualization of the right ICA from its origin to the intracranial portion (arrow); d: three-dimensional MRA reconstruction demonstrating absent right ICA flow with preserved contralateral and vertebrobasilar vessels.

Carotid Doppler ultrasonography revealed complete occlusion of the right ICA without a flow signal. MRA showed no opacification of the right ICA along its cervical and intracranial segments, confirming total occlusion. The left ICA and vertebrobasilar circulation were well visualized and patent. Collateral circulation was noted through the circle of Willis, contributing to partial compensation of the right-sided flow deficit. These findings were consistent with chronic right ICA occlusion without evidence of reconstitution in distal segments (Figure 2).

**Figure 3:**
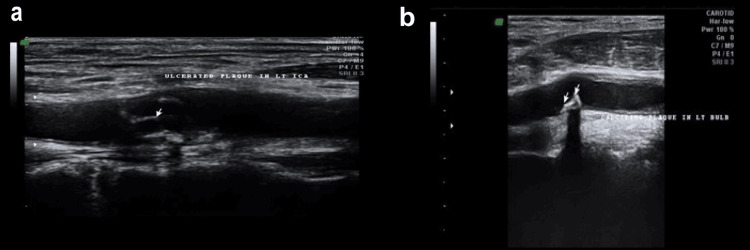
Ultrasonographic images showing ulcerated atherosclerotic plaques in the left carotid artery a: longitudinal B-mode ultrasonographic image demonstrating an ulcerated plaque in the left internal carotid artery (ICA), the plaque shows an irregular surface with a distinct ulcer crater (arrow), indicating plaque rupture; b: transverse sonographic image showing an ulcerated plaque in the left carotid bulb, the echolucent area within the plaque (arrows) represents the ulcer cavity with disrupted fibrous cap margins.

On carotid ultrasonography, ulcerated atherosclerotic plaques were observed in the left carotid artery in selected patients. Figures 3a, 3b demonstrate typical imaging features of plaque ulceration. In Figure 3a, the longitudinal section of the left ICA reveals an irregularly contoured atherosclerotic plaque with a focal depression consistent with plaque ulceration. In Figure 3b, the transverse section of the left carotid bulb displays a distinct ulcer cavity within the plaque surface, appearing as an anechoic recess extending into the plaque core. These findings are characteristic of unstable or vulnerable plaques, indicating a higher risk of embolic cerebrovascular events.

**Figure 4:**
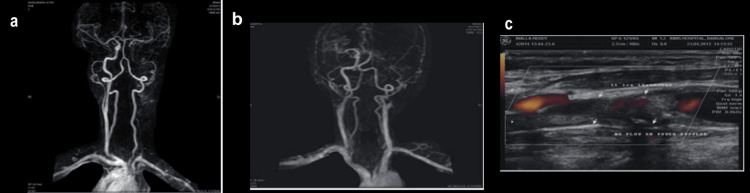
Imaging findings demonstrating carotid artery stenosis and occlusion a, b: magnetic resonance angiography (MRA) of the neck vessels showing complete occlusion of the left internal carotid artery (ICA) with absence of flow-related enhancement distal to the carotid bifurcation, collateral flow through the circle of Willis is visualized; c: the Color Doppler ultrasound image demonstrates the absence of flow in the left ICA in power Doppler mode, confirming complete occlusion, the lumen appears echogenic and filled with thrombotic material (arrows), consistent with a chronic occlusive plaque.

MRA and carotid Doppler imaging revealed significant vascular pathology in the left carotid system. MRA demonstrated complete occlusion of the left ICA, evidenced by non-visualization of the arterial lumen beyond the bifurcation and absence of normal flow-related signal intensity (Figures 4a, 4b). Collateral circulation was observed through the contralateral carotid and vertebrobasilar systems. Complementary Doppler ultrasonography (Figure 4c) confirmed the lack of flow in the left ICA on power Doppler interrogation, with echogenic material occupying the lumen, suggesting an organized thrombus or chronic occlusive plaque. These combined findings confirmed complete occlusion of the left ICA with compensatory collateral perfusion.

## Discussion

This observational correlation study aimed to assess the diagnostic efficacy of Doppler sonography in evaluating extracranial carotid and vertebral artery pathology in stroke patients, and to compare its findings with those of MRA. The results demonstrate that Doppler and MRA effectively identify significant vascular abnormalities, particularly in the ICA and vertebral arteries, most commonly associated with ischemic stroke.

Our study showed complete concordance between Doppler and MRA in detecting pathologies of the ICA and vertebral arteries (56% and 52%, respectively; p=1.000). These findings are consistent with the meta-analysis by Saxena et al. [7], which reported that Doppler achieved approximately 86% sensitivity and 87-90% specificity for ≥70% stenosis, while MRA demonstrated around 95% sensitivity and 90% specificity. Nederkoorn et al. [8] similarly reported high performance in detecting occlusions, with Doppler showing sensitivity ranging from 96% to 98%, and specificity of 100%, while MRA demonstrated both sensitivity and specificity between 98% and 100%. This supports our findings of comparable accuracy in detecting total occlusions and fresh thromboses in the ICA and vertebral arteries (52%).

In our study, Doppler missed several high-grade stenoses (80-99%) detected by MRA, including 4% at the carotid bulb and 2% at the ICA, underscoring Doppler’s resolution limitations. In the 70-79% stenosis range, Doppler and MRA showed complete agreement (4% at the bulb and 2% at the ICA), reinforcing the reliability of Doppler in identifying moderately high-grade stenoses when appropriate velocity windows are available. According to Muto et al. [9], MRA often overestimates stenosis compared to Doppler in severe cases, but provides superior visualization when the degree of narrowing exceeds 75%. Mittl et al. [10] reported that MRA demonstrated a sensitivity of approximately 92.4% and specificity of 74.5% for stenoses between 70% and 99%, whereas Doppler performed less favorably. These findings support our observation that Doppler may underdetect certain critical stenoses. Our study also found that MRA identified collaterals in 30% of ICA and 34% of vertebral artery cases, whereas Doppler failed to detect any. This underscores MRA’s superior capability in visualizing collateral networks. These findings align with preclinical studies using high-resolution dynamic susceptibility contrast (DSC)-MRI, highlighting the importance of assessing collateral supply in predicting stroke outcomes. Automated collateral scoring has also demonstrated MRA’s ability to visualize perfusion patterns [11]. The statistically significant difference (p < 0.001) in collateral detection between the two modalities further emphasizes MRA’s enhanced sensitivity to perfusion routes critical to stroke recovery.

In our study, Doppler overestimated moderate carotid bulb stenosis in the 50-69% range (4%), a finding not confirmed by MRA. This discrepancy may be explained by the operator-dependent nature of Doppler and the complex vascular anatomy, particularly in tortuous segments or regions with turbulent flow. A 2019 study by Ismail et al. [12] proposed consensus-based Doppler thresholds to improve the detection of mild stenosis. Huston’s cohort [12] reported Doppler sensitivity of approximately 86% for this range, while MRA demonstrated greater efficacy for stenoses ≥70%.

Both modalities detected a single case of complete CCA occlusion, highlighting the rarity, but detectability, of CCA disease. The substantial concordance in detecting occlusions and thromboses supports the value of Doppler as a frontline tool in acute settings. Current stroke guidelines recommend Doppler as a rapid bedside screening method, and studies such as Heijenbrok-Kal et al. [13] have reported a sensitivity of 95% and specificity of 99% for occlusions.

The limitations observed in our single-center cohort of 150 patients are consistent with broader critiques in the existing literature. Meta-analyses comparing Doppler ultrasound, MRA, and digital subtraction angiography (DSA) highlight the superiority of MRA in the evaluation of vascular stenosis, while also emphasizing Doppler sonography’s susceptibility to operator dependency. In the present study, Doppler examinations were performed by experienced operators; however, inter-observer variability and formal agreement analyses were not assessed, which represents an additional limitation. Furthermore, the absence of a gold-standard DSA reference limits definitive validation of imaging findings. Despite these limitations, supported by existing literature, our study demonstrates that Doppler sonography remains a reliable and cost-effective modality for the detection of vascular occlusions and thromboses, whereas MRA provides superior accuracy in identifying high-grade stenoses and in visualizing collateral circulation. Taken together, the two imaging techniques serve as complementary diagnostic tools, balancing accessibility, rapid assessment, and diagnostic precision.

## Conclusions

This study demonstrates that Doppler sonography and MRA are both practical, noninvasive imaging modalities for the evaluation of extracranial carotid and vertebral artery pathology in patients presenting with stroke or TIA. Doppler sonography showed concordant findings with MRA in the identification of complete vascular occlusions and thromboses, supporting its role as a rapid and accessible first-line imaging technique in acute clinical settings. However, MRA consistently demonstrated superior capability in delineating high-grade stenosis and collateral circulation, particularly within the internal carotid and vertebral arteries, which were less reliably visualized on Doppler examination. These observations highlight the known operator dependency and spatial resolution limitations of Doppler sonography, especially in anatomically complex regions such as the carotid bulb. While Doppler remains a cost-effective and widely available modality, its performance in characterizing moderate-to-severe stenosis appears variable in the absence of a reference standard. In contrast, although MRA is more resource-intensive, it provides more consistent anatomical detail and vascular characterization. Within this context, the complementary use of both modalities may be clinically informative for vascular assessment; however, this interpretation is inferential and not based on outcome or management analyses in the present study. Further investigations incorporating digital subtraction angiography as a reference standard and correlating imaging findings with clinical outcomes are warranted to substantiate these observations.
